# Antiviral and Virucidal Activities of Camptothecin on Fowl Adenovirus Serotype 4 by Blocking Virus Replication

**DOI:** 10.3389/fcimb.2022.823820

**Published:** 2022-04-14

**Authors:** Dongdong Yin, Lei Yin, Jieru Wang, Xuehuai Shen, Yin Dai, Ruihong Zhao, Xiaomiao Hu, Hongyan Hou, Danjun Zhang, Guijun Wang, Kezong Qi, Xiaocheng Pan

**Affiliations:** ^1^ Anhui Province Key Laboratory of Livestock and Poultry Product Safety Engineering, Livestock and Poultry Epidemic Diseases Research Center of Anhui Province, Institute of Animal Husbandry and Veterinary Science, Anhui Academy of Agricultural Sciences, Hefei, China; ^2^ Anhui Province Key Laboratory of Veterinary Pathobiology and Disease Control College of Animal Science and Technology, Anhui Agricultural University, Hefei, China

**Keywords:** fowl adenovirus serotype 4, camptothecin, antiviral activity, virus replication, hepatitis-hydropericardium syndrome

## Abstract

Fowl adenovirus serotype 4 (FAdV-4) caused hepatitis–hydropericardium syndrome in poultry and caused huge economic losses to the poultry industry. At present, antiviral drugs have not been reported to be effective against this virus, and new treatment methods are urgently needed to treat FAdV-4. Camptothecin has been shown to have antiviral activity against various viruses; however, whether it can inhibit FAdV-4 infection remains unclear. This study aimed to explore the anti-FAdV-4 effects and mechanisms of camptothecin *in vitro* and *in vivo*. Several camptothecin treatments were used to study the antiviral activity of camptothecin on FAdV-4-infected Leghorn male hepatocellular (LMH) cells. The FAdV-4 titers of mock and camptothecin-treated infected cell cultures were determined using tissue culture infective dose assay, and the FAdV-4 copy number was determined using quantitative real-time polymerase chain reaction. In addition, the therapeutic effect of camptothecin on FAdV-4-infected chickens was also evaluated. The results showed that camptothecin significantly reduced the viral replication in LMH cells in a dose-dependent manner, resulting in a reduction in viral titer, viral copy number, and viral Hexon protein expression. Camptothecin was also found to have a significant inhibitory effect on the viral replication step. Finally, camptothecin showed anti-FAdV-4 efficacy in the chicken infection model, and the survival rate was improved. This study was novel in proving that camptothecin had a protective effect against FAdV-4, indicating its potential as an antiviral drug against FAdV-4 infection.

## Introduction

The hepatitis–hydropericardium syndrome (HHS) is an important infectious disease of poultry caused by fowl adenovirus serotype 4 (FAdV-4) (Li et al., 2017; Park et al., 2017). HHS was first reported in broiler flocks in Pakistan in 1987 (Cheema et al., 1989). Since 2015, outbreaks of HHS happened in several provinces in China, including Shandong, Henan, Fujian, and Shaanxi, and the characteristic lesions of affected poultry were enlarged liver with scattered hemorrhagic spots on the surface, and a large amount of straw-yellow fluid in the pericardial cavity, with a mortality rate of 30%–80% (Liu et al., 2016; Park et al., 2017; Xia et al., 2017). FAdV-4 has a wide range of hosts, infecting not only laying hens and broilers but also ducks, geese, and a variety of wild birds. The diversity of hosts infected by FAdV-4 increases the potential risk of cross-host transmission, making the scientific prevention and control of the disease more difficult (Li et al., 2017; Yu et al., 2018; Wei et al., 2019). In addition, FAdV-4 can be transmitted not only horizontally by the fecal-oral route but also vertically through chicken embryos, posing a threat to the production of chicken embryo–derived veterinary vaccines (Li et al., 2017; Li et al., 2019). Thus, developing antiviral drugs effective reduce FAdV-4 replication is an urgent need.

Camptothecin is a plant alkaloid with antiviral and antitumor activities (Montoro et al., 2010; Liu et al., 2015; Martino et al., 2017). It has antiviral activity against a variety of viruses, including adenovirus type 2 (ADV2) (Horwitz and Brayton, 1972), herpes simplex virus 2 (HSV-2) (Li et al., 2002; Zhou et al., 2018), and enterovirus 71 (EV71) (Wu and Chu, 2017). Horwitz et al. have shown that camptothecin is an effective ADV2 replication inhibitor (Horwitz and Brayton, 1972). Wu et al. reported that camptothecin could hinder the transcription and translation of EV71 RNA (Wu and Chu, 2017). However, the antiviral effect of camptothecin against FAdV-4 has not been investigated, and the underlying mechanisms remain to be elucidated.

In this study, the antiviral activity of camptothecin against FAdV-4 in Leghorn male hepatocellular (LMH) cells was first explored. Furthermore, the therapeutic effects of this drug in chickens were investigated. All these findings suggested that camptothecin might be a candidate inhibitor for effective control of FAdV-4 infection.

## Materials and Methods

### Cells, Viruses, and Reagents

The LMH cells (ATCC CRL-2117) were cultured in Dulbecco’s modified Eagle medium (DMEM)/F12 (Gibco, USA) supplemented with 10% fetal bovine serum (FBS) (Gibco), 100 U/mL penicillin, and 0.1 mg/mL streptomycin (Beyotime, China) in a 37°C incubator supplied with 5% CO_2_. The FAdV-4 AH-F19 strain (Accession number: MN781666) was isolated from a laying flock and propagated in the laboratory (Yin et al., 2020). Camptothecin with purity >99% was provided by PureOne Biotechnology in Shanghai, China. It was dissolved in dimethyl sulfoxide (DMSO, Solarbio, China) and stored at 4°C.

### Cytotoxic Effect of Camptothecin

The cytotoxicity of camptothecin in LMH cells was assayed using a Cell Counting Kit-8 (CCK-8, Beyotime, China) to eliminate the interference of the camptothecin concentration on the change of cellular growth. The LMH cells were seeded in 96-well plates at a density of 1 × 10^4^ cells/well and incubated at 37°C. Once the LMH cells formed a monolayer, the media was discarded and replenished with a fresh medium containing increasing concentrations of camptothecin (0μM, 0.1μM, 0.5μM, 1μM, 5μM, 10μM, and 50μM). After 48 h of incubation, 10 μL of CCK-8 solution was added to each well and incubated at 37°C for 1 h. Subsequently, the absorbance rates were measured at 450 nm using a microplate reader (Thermo Scientific, USA).

### Camptothecin Treatment and FAdV-4 Infection *In Vitro*

The LMH cells were seeded in a 12-well plate to investigate the antiviral effect of camptothecin on FAdV-4. Serially diluted camptothecin (0μM, 0.1μM, 0.5μM, 1μM, 5μM, and 10μM) was added to the culture medium of the LMH cells. DMSO (final concentration was 0.1%) was used as the negative control. FAdV-4 [1 multiplicity of infection (MOI)] was then added to the cells, and cultures were incubated for 48 h at 37°C. An indirect immunofluorescence assay (IFA) was used to examine the effect of camptothecin on FAdV-4 infection in LMH cells. The cells were then fixed with 4% paraformaldehyde for 15 min, washed with phosphate-buffered saline (PBS), and then permeabilized with 0.1% Trition X-100 (Sangon Biotech, China) for 15 min at 37°C. The treated cells were incubated with the anti-Hexon monoclonal antibody prepared in the laboratory (1:200 dilution) for 2 h at 37°C. The cells were then washed three times with PBS and incubated with fluorescein isothiocyanate–labeled goat anti-mouse immunoglobulin (IgG) (H + L) antibody (1:500 dilution) (Beyotime, China) at 37°C for 30 min. Nuclei were stained with 1 mg/mL 4′, 6-diamidino-2-phenylindole (DAPI, Beyotime, China). The samples were observed under an inverted fluorescence microscope (Olympus, Japan).

### Virus Titer

The LMH cells were seeded into 96-well plates. The virus was serially diluted with DMEM/F12 from 10^-1^-fold to 10^-8^-fold containing 2% FBS and added to corresponding wells. Then, the culture plates were incubated at 37°C in the presence of 5% CO_2_. The cytopathic effects were observed under the microscope, and the values of median tissue culture infectious dose (TCID_50_) were calculated using the Reed and Muench method (Reed and Muench, 1938).

### Quantitative Polymerase Chain Reaction

The FAdV-4 copy number was determined by quantitative polymerase chain reaction (qPCR) as previously described (Wang et al., 2019). Total DNA was extracted with TIANamp Virus DNA/RNA Kit (TIANGEN, China) following the manufacturer’s protocols. The FAdV-4 *Hexon* gene was used as an indicator for the presence of viral DNA. To generate the FAdV-4 DNA standard curve, the target sequence of *Hexon* gene was cloned into the pMD-19T easy vector to create a pMD-19T-Hexon plasmid. Copy numbers of the viral DNAs were calculated by comparison of the standard curve based on the positive template of the pMD-19T-Hexon plasmid. All primers for qPCR used in this study were designed using the Primer 5 software (**Table S1**) and synthesized by Sangon Biotech (Shanghai, China).

### Western Blot Analysis

The cells were treated with lysis buffer, the supernatant was collected by centrifugation, and the protein concentration was measured using a bicinchoninic acid Protein Assay Kit (Thermo Fisher Scientific, USA). The cell lysates were subjected to 10% sodium dodecyl sulfate–polyacrylamide gel electrophoresis and then transferred onto polyvinylidene fluoride (Millipore, USA) membranes by semidry blotting (Bio-Rad Trans-Blot Turbo System, USA). The membranes were blocked in Tris-buffered saline containing 5% nonfat dry milk for 2 h at 37°C and then washed with PBS containing 0.1% Tween-20. Protein was blotted with different antibodies. The anti-Hexon monoclonal antibody was diluted at 1:500, and horseradish peroxidase–conjugated anti-mouse IgG (Abmart, China) was diluted at 1:5000. The membranes were treated with enhanced chemiluminescence (Beyotime, China). The digital imaging was performed using a Tanon 5200 Chemi-Image System (Biotanon, China).

### Virus Attachment Assay

The LMH cells were seeded in a 6-well plate and incubated at 37°C. When the LMH cells formed a monolayer, the 6-well plate was placed at 4°C for 1 h, then the media was discarded, and the cells were washed three times with ice-cold PBS. The LMH cells were pretreated with camptothecin (10μM) or DMSO for 1 h at 37°C and then infected with FAdV-4 (1 MOI) for the time indicated (30 min and 1 h) at 4°C, shake once in 15min (**Figure 3A**). The cells were then washed with ice-cold PBS, and the FAdV-4 DNA levels in the cells were measured using qPCR.

### Virus Entry Assay

The LMH cells were seeded in a 6-well plate and incubated at 37°C. When the LMH cells formed a monolayer, the 6-well plate was placed at 4°C for 1 h, then the media was discarded, and the cells were washed three times with ice-cold PBS. The LMH cells were infected with FAdV-4 (1 MOI) at 4°C for 1 h. The supernatant was replaced with DMEM/F12 containing camptothecin (10μM) or DMSO and then incubated at 37°C for the time indicated (1 h and 2 h) (**Figure 3B**). The cells were washed with PBS (pH 3) to remove the non-internalized virus. The FAdV-4 DNA levels in the cells were measured using qPCR.

### Virus Replication Assay

The LMH cells were incubated with FAdV-4 (1 MOI) at 37°C for 1 h and washed three times with PBS to remove the free virus. At 4 hpi, the culture medium was replaced with fresh DMEM/F12 containing camptothecin (10μM) or DMSO, and the cultures were incubated at 37°C (**Figure 3C**). The FAdV-4 DNA in the samples collected at 8 and 10 hpi were measured using qPCR.

### Virus Release Assay

The LMH cells were infected with FAdV-4 (1 MOI) for 1 h at 37°C. The culture medium was then replaced with fresh DMEM/F12. At 10 hpi, the cells were washed three times with PBS and the culture medium was replaced with fresh DMEM/F12 containing camptothecin (10μM) or DMSO. The cultures were incubated at 37°C for 1 h and 2 h, and the supernatants were then harvested (**Figure 3D**). The FAdV-4 DNA levels in the cells were measured using qPCR and Hexon protein was detected by Western blot.

### Infection of Chickens

Forty, 3-week-old specific-pathogen-free chickens were used in this experiment. The chickens were randomly divided into 4 groups (10 per group). In the FAdV-4 group, chickens were intramuscularly injected with FAdV-4 (10^5.0^ TCID_50_ doses per chicken). In the control group, all chickens were intramuscularly injected with the same dosage of sterile PBS containing DMSO (final concentration was 0.1%) as a negative control group. In the FAdV-4 + camptothecin (10 mg/kg) and FAdV-4 + camptothecin (20 mg/kg) groups, chickens in each group were intramuscularly injected with camptothecin to the concentration described. Treatment was started 4 h after viral infection. When the chickens died, the liver and kidney samples were collected for histopathological examination and qPCR. At 2 weeks after infection, all surviving chickens were euthanized, following which different tissues were collected and dealt as previously mentioned. The animal experiments were approved by the Committee on the Ethics of Animal Care and Use at Anhui Academy of Agricultural Sciences (Anhui, China).

### Statistical Analysis

All data were tested for significance using the Student *t* test or one-way analysis of variance. Data were analyzed using GraphPad Prism Software 8.0 (USA). A *P* value <0.05 indicated a statistically significant difference.

## Results

### Dose-Dependent Inhibitory Effect of Camptothecin on FAdV-4

**Figure 1:**
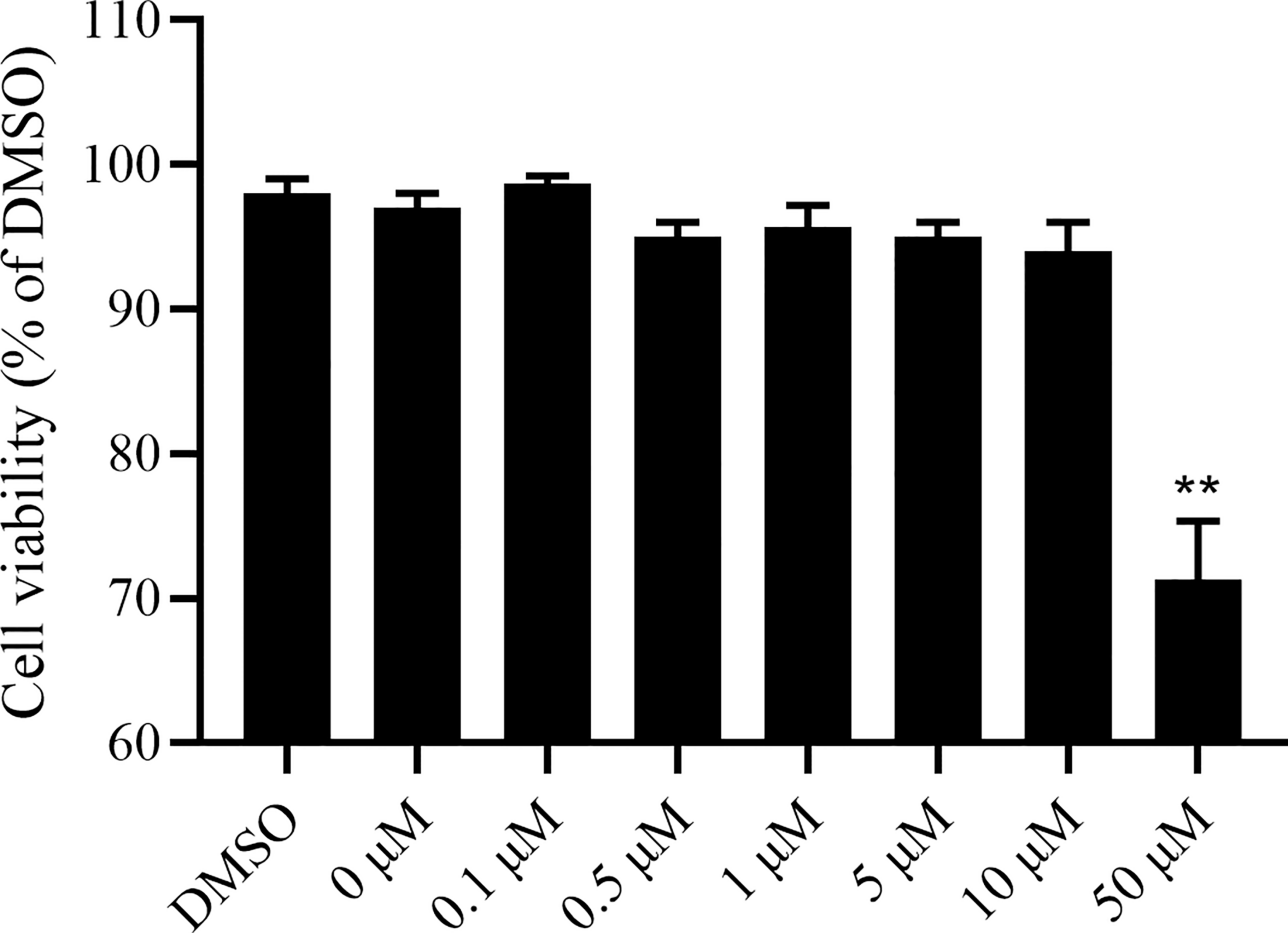
Camptothecin cytotoxic effect on LMH cells. LMH cells were incubated with camptothecin at various doses ranging from 0μM to 50μM for 48 h. Then, CCK-8 assays were performed to examine the effect of camptothecin on the viability of LMH cells. All experiments were performed in triplicate (*n* = 3). The error bars indicate the standard deviation of data from three independent experiments (**P < 0.01).

In this study, the CCK-8 colorimetric method was first used to detect the toxicity of camptothecin to LMH cells. The LMH cells were treated with camptothecin at increasing concentrations of 0.1μM, 0.5μM, 1μM, 5μM, 10μM, and 50μM, and the cytotoxicity was evaluated after 48 h of incubation. Compared with the untreated group, 0.1μM, 0.5μM, 1μM, 5μM, and 10μM camptothecin had no obvious cytotoxic effect on LMH cells, while the LMH cells treated with 50μM camptothecin showed a significant inhibitory effect on proliferation (**Figure 1**). Therefore, for subsequent experiments, the optimal concentration of camptothecin was recommended to be 10μM.

**Figure 2:**
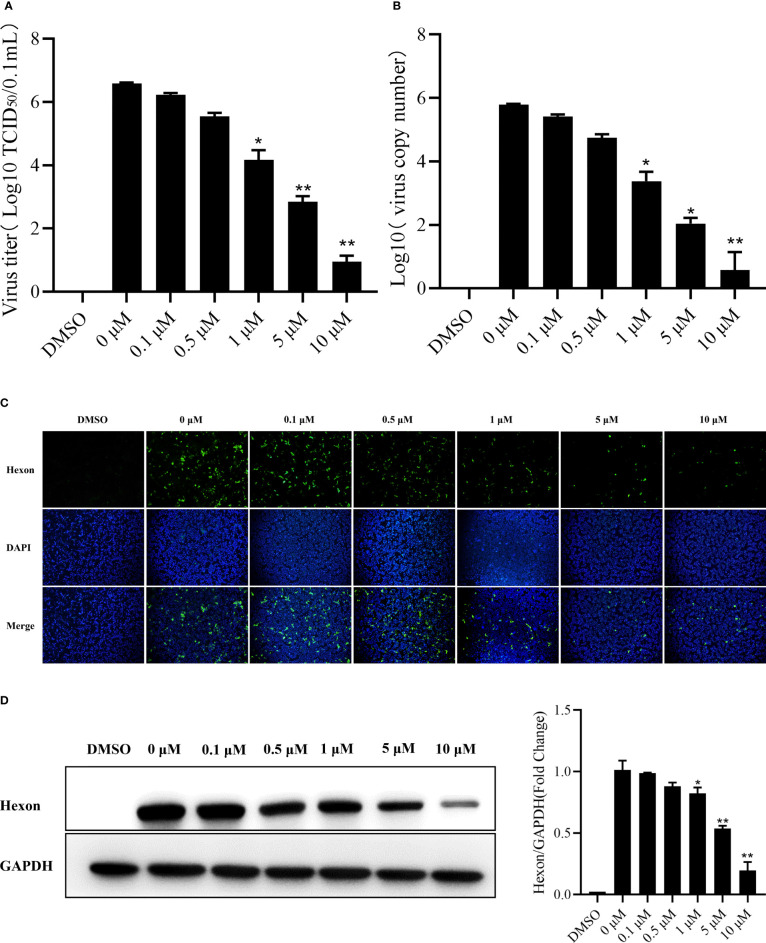
Dose-dependent inhibition of FAdV-4 infection by camptothecin. LMH cells were treated with different concentrations of camptothecin (0μM, 0.1μM, 0.5μM, 1μM, 5μM, and 10μM) at 37°C and subsequently infected with FAdV-4 at an MOI of 1 for 48 h **(A)** Virus titers were determined by measuring the TCID_50_ assay. **(B)** Virus copy numbers were quantified by qPCR. **(C)** FAdV-4 Hexon protein expression in LMH cells was detected using indirect immunofluorescence staining. **(D)** FAdV-4 Hexon protein expression in cells was detected using Western blot analysis. All experiments were performed in triplicate (*n* = 3). Error bars represent the standard deviation. The asterisks in the figures indicate significant differences (^*^ *P* < 0.05; ^**^ *P* < 0.01).

The LMH cells were treated with camptothecin (0μM, 0.1μM, 0.5μM, 1μM, 5μM, and 10μM) to study whether camptothecin had an antiviral effect on FAdV-4, and we determined the virus replication inhibition in cells treated. As shown in **Figures 2A, B**, camptothecin reduced the virus titer and FAdV-4 copy number of LMH cells in a dose-dependent manner. An IFA was used to detect the effects of camptothecin on the expression of the viral Hexon protein in LMH cells. Camptothecin dose-dependently inhibited the expression of the Hexon protein in LMH cells (**Figure 2C**). A similar result in the expression of Hexon protein was obtained *via* Western blot analysis (**Figure 2D**). All these data indicated that camptothecin had an inhibitory effect on FAdV-4 replication.

### Effect of Camptothecin on Viral Attachment, Entry, Replication, and Release

**Figure 3:**
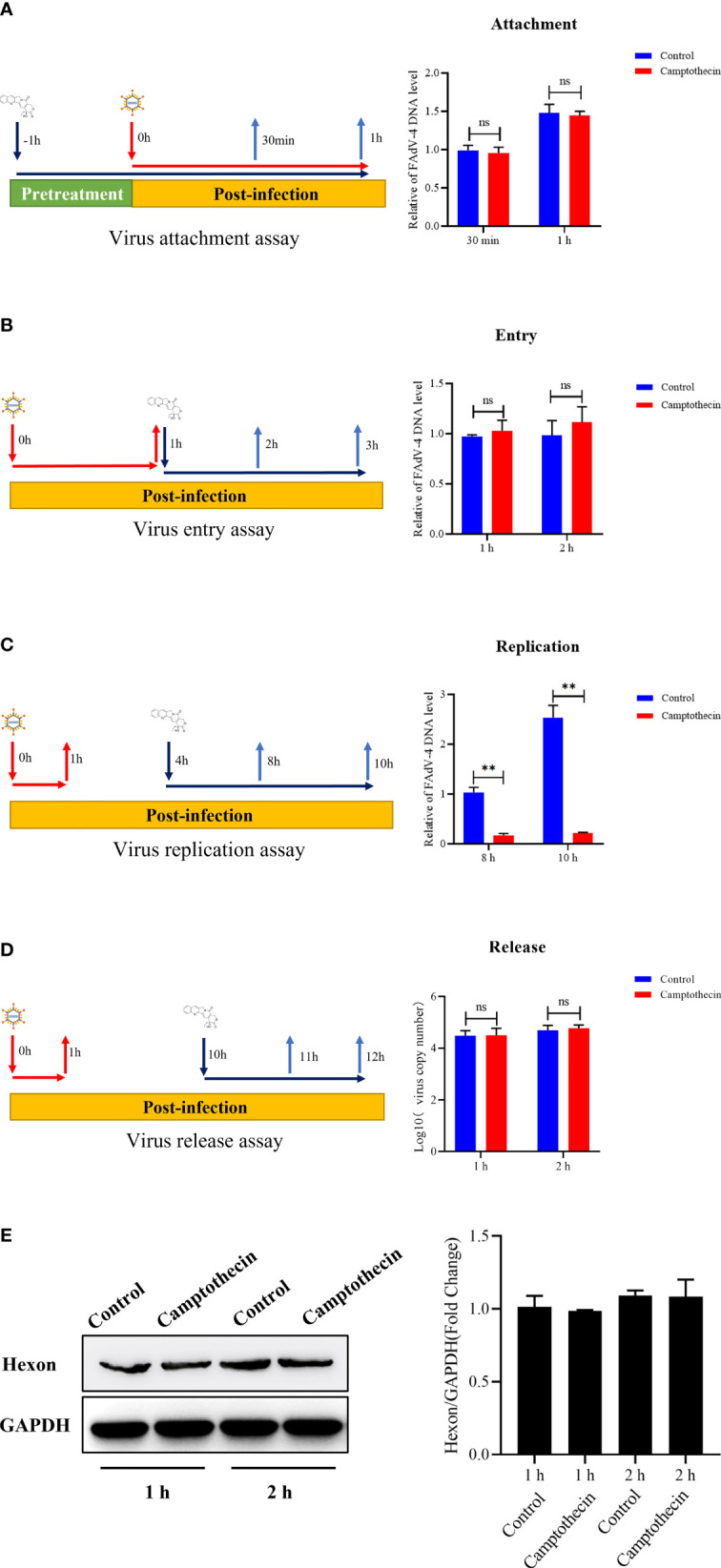
Effect of camptothecin on the attachment, entry, replication, and release of FAdV-4. FAdV-4 infected LMH cells and added camptothecin in different life cycles of the virus. The FAdV-4 DNA levels in LMH cells were measured using qPCR. **(A)** Virus attachment assay. **(B)** Virus entry assay. **(C)** Virus replication assay. **(D, E)** Virus release assay. All experiments were performed in triplicate (*n* = 3). Error bars represent the standard deviation. The asterisks in the figures indicate significant differences (^*^ *P* < 0.05; ^**^ *P* < 0.01; ns, not significant).

The qPCR was used to detect the FAdV-4 DNA levels to determine the effect of camptothecin on FAdV-4 attachment, entry, replication, and release to further explore the mechanism of camptothecin-inhibiting FAdV-4 infection. The results showed that before FAdV-4 infection, camptothecin treatment did not significantly block FAdV-4 attachment to LMH cells, indicating that camptothecin does not inhibit FAdV-4 attachment to cells (**Figure 3A**). As shown in **Figure 3B**, when evaluating the effect of camptothecin on FAdV-4 entry, camptothecin treatment accompanied by viral entry did not significantly impede virus replication compared with the treatment with DMSO. Then, the effect of camptothecin on FAdV-4 replication was examined by adding camptothecin in the virus replication stage. As shown in **Figure 3C**, camptothecin treatment significantly reduced FAdV-4 DNA levels compared with treatment with DMSO, indicating that camptothecin inhibited FAdV-4 replication. In addition, the virus release assay showed no significant difference in the levels of FAdV-4 copy number and Hexon protein levels in the treated with camptothecin and DMSO (**Figures 3D, E**). Taken together, these results indicated that camptothecin mainly inhibited FAdV-4 infection by affecting virus replication.

### Camptothecin Inhibited FAdV-4 Infection *In Vivo*

**Figure 4:**
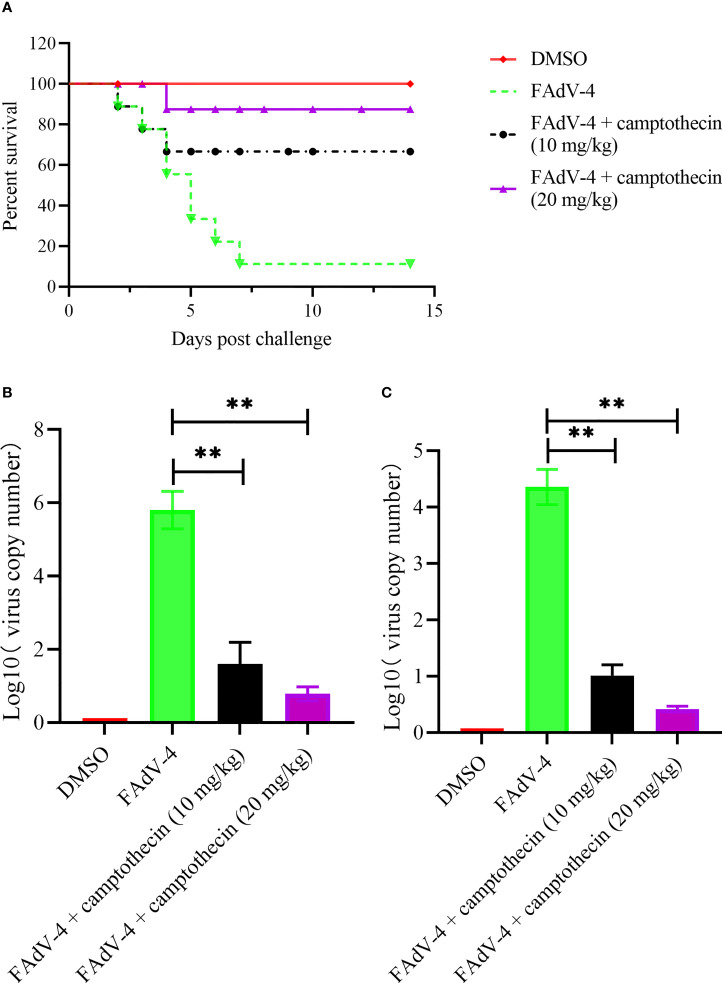
Camptothecin inhibited FAdV-4 infection *in vivo*. Chickens were infected with FAdV-4 at 4 h before the beginning of the camptothecin treatment. **(A)** Survival rate was measured for 2 weeks. **(B, C)** Virus copy numbers in the liver and kidney were detected using qPCR in all chicken groups. All experiments were performed in triplicate (*n* = 3). Error bars represent the standard deviation. The asterisks in the figures indicate significant differences (^**^ *P* < 0.01).

After 4 h of viral infection, chickens were treated with camptothecin to evaluate the inhibitory effect of camptothecin on FAdV-4 infection *in vivo*. After 2 weeks, the survival rate of the animals was measured, and the therapeutic effect of camptothecin was evaluated. As shown in **Figure 4A**, the survival rate of infected chickens treated with 10 and 20 mg/kg camptothecin was significantly improved compared with the control group. Compared with the control group, the viral copy number in the liver and the kidney of chickens infected with FAdV-4 increased significantly. In contrast, the number of virus copies in different tissues of chickens in the camptothecin treatment group was significantly lower than that in the FAdV-4 group (**Figures 4B, C**).

**Figure 5:**
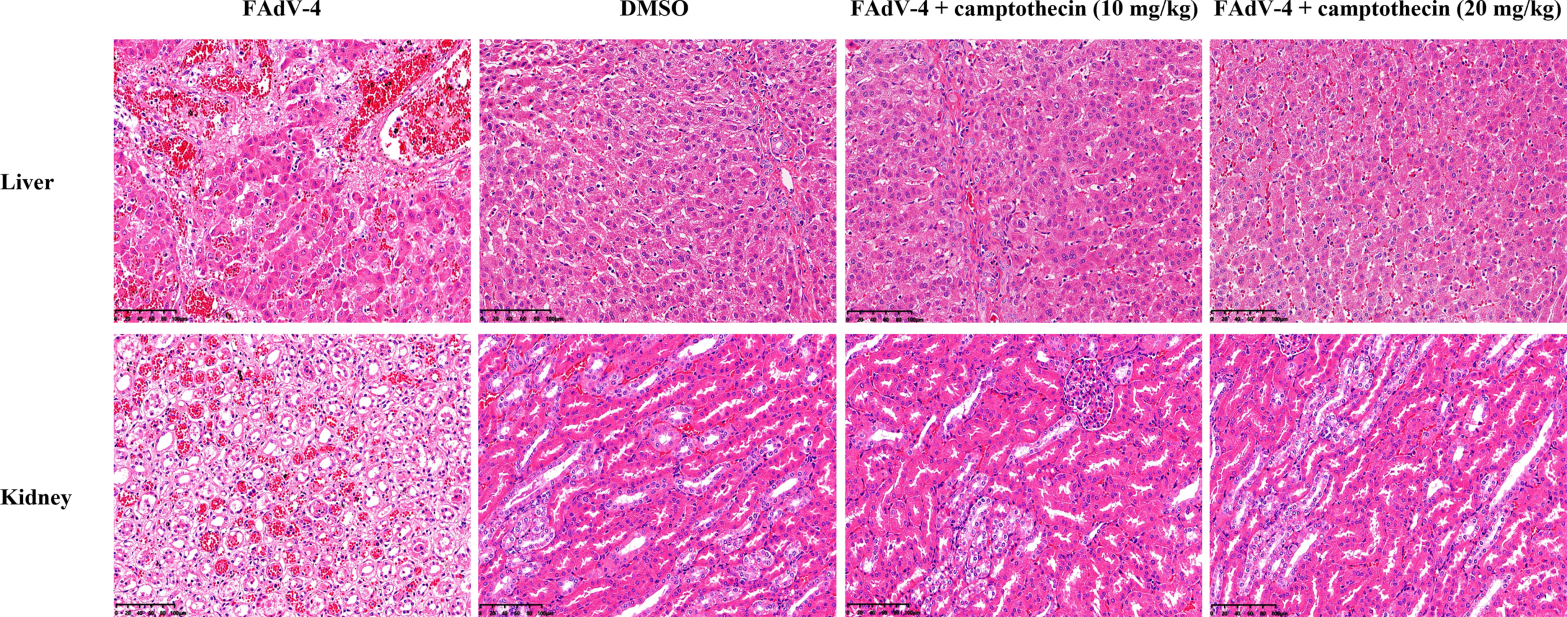
Histopathologic lesions of the liver and kidney after camptothecin treatment in FAdV-4-infected chickens. Histopathologic changes (200×) of the liver and kidney tissues obtained from chickens.

The histopathological changes of the liver and kidney of each group of chickens were further analyzed. FAdV-4 infection caused fatty degeneration of liver cells, liver sinusoids were full of red blood cells, and basophilic nuclear inclusions appeared in the liver cells (**Figure 5**), which was a typical histopathological change of HHS. On the contrary, the liver of FAdV-4-infected chickens treated with camptothecin showed only slight pathological changes, which indicated that camptothecin treatment protected the liver of the chickens from the damage caused by FAdV-4 infection (**Figure 5**). As for the kidney, after FAdV-4 infection, hematoxylin and eosin staining showed renal vasodilation, congestion, necrosis of renal tubular epithelial cells, condensation and fragmentation of nuclei, and the lesions in the high- and low-dose groups of camptothecin were significantly reduced (**Figure 5**). The aforementioned results indicated that camptothecin had a protective effect on FAdV-4-infected chickens.

## Discussion

The outbreak of HHS has caused a heavy economic burden on the poultry industry. Research is currently being conducted for the development of vaccines to prevent this disease, but no antiviral drugs effective against the virus have been reported. Therefore, the development of an effective antiviral drug for treating FAdV-4 infection is urgently needed. Natural products have always been a rich and diverse resource for drug discovery, and one of the advantages is that their organic source makes them more likely to be biologically active (Martinez et al., 2015). Camptothecin is a natural compound extracted from the bark of *Camptotheca acuminata*. It has the advantages of low toxicity, high efficiency, and easy availability of materials (Wall et al., 1966). In the present study, camptothecin was shown to have a significant inhibitory effect on FAdV-4 infection *in vitro* and *in vivo*. Camptothecin exhibits antiviral activity against FAdV-4 by inhibiting viral replication. The results of this study indicated that camptothecin may be used as an effective antiviral drug for treating FAdV-4 infection. Increasing evidence showed that camptothecin had antiviral effects, including ADV2, HSV-1, and EV71. However, the antiviral activity of camptothecin on FAdV-4 and its mechanism have not been studied. In this study, the results indicated that camptothecin had a dose-dependent antiviral effect on FAdV-4, which manifested in reducing virus titer, virus copy number, and Hexon protein expression. In addition, camptothecin could also inhibit virus replication in different tissues of FAdV-4-infected chicken and improve the survival rate of FAdV-4-infected chicken. Therefore, the results of this study indicated that camptothecin treatment was effective against FAdV-4 infection *in vivo* and *in vitro*.

The viral life cycle includes attachment to target cells, internalization of the virus, and replication and release from target cells. Many antiviral drugs work by blocking one or more of these steps (Liu and Thorp, 2002). For instance, Zhu et al. showed that epigallocatechin gallate had antiviral activity against the duck Tembusu virus by inhibiting virus attachment, receptor binding, and entry (Zhu et al., 2021). Wang et al. discovered that tomatine reduced the infection of the porcine epidemic diarrhea virus by preventing the virus from replication (Wang et al., 2020). Tsai showed that tryptanthrin prevented the early and late stages of human coronavirus NL63 (HCoV-NL63) replication by blocking viral genome synthesis (Tsai et al., 2020). Several reports showed that camptothecin could inhibit viral infection through a variety of potential mechanisms. For example, camptothecin inhibited DNA synthesis and caused pre-formed viral DNA fragmentation in the cell to hinder ADV2 replication (Horwitz and Brayton, 1972). It prevented EV71 virus RNA replication and protein synthesis by inhibiting DNA topoisomerase 1 (Wu and Chu, 2017). The additional time measurement could provide a preliminary understanding of the infection stage of the action of camptothecin. This study indicated that the antiviral effect of camptothecin on FAdV-4 may be achieved by inhibiting virus replication.

In conclusion, camptothecin exhibited the potential of antiviral activity against FAdV-4 *via* significantly inhibiting virus replication. These results provided support for camptothecin as an effective drug candidate for the treatment and control of FAdV-4, and it is worthwhile to study further the application of camptothecin in treating FAdV-4. In addition, this study showed that different doses of camptothecin were effective in chickens. Still, the safety, pharmacokinetics, and efficacy of camptothecin for treating FAdV-4 need to be further evaluated in chickens.

## Data Availability Statement

The original contributions presented in the study are included in the article/**Supplementary Material**. Further inquiries can be directed to the corresponding author.

## Ethics Statement

The animal study was reviewed and approved by the Committee on the Ethics of Animal Care and Use at Anhui Academy of Agricultural Sciences (Anhui, China).

## Author Contributions

Among the authors, DY and LY performed the experiments and wrote the paper. JW, XS and YD were responsible for data statistics and analysis. DZ, RZ, XH and HH were responsible for editing the paper. XP, GW, and KQ conceived and designed the experiments. All authors contributed to the article and approved the submitted version.

## Funding

This study was supported financially by the Major Science and Technology Special Project in Anhui Province (No. 202003a06020012); the Anhui Academy of Agricultural Sciences Platform Project (No. 2021YL065), and the Anhui Province Poultry Industry Technology System (No. AHCYJSTX-06).

## Conflict of Interest

The authors declare that the research was conducted in the absence of any commercial or financial relationships that could be construed as a potential conflict of interest.

## Publisher’s Note

All claims expressed in this article are solely those of the authors and do not necessarily represent those of their affiliated organizations, or those of the publisher, the editors and the reviewers. Any product that may be evaluated in this article, or claim that may be made by its manufacturer, is not guaranteed or endorsed by the publisher.
