# Supplementary material for: Antiviral and Virucidal Activities of Camptothecin on Fowl Adenovirus Serotype 4 by Blocking Virus Replication
Source: Front Cell Infect Microbiol. 2022 Apr 14;12:823820. doi: 10.3389/fcimb.2022.823820 (PMC9046556; doi:10.3389/fcimb.2022.823820)
Supplement: Supplementary file 1 [file Table_1.docx]

| Primer name | Sequence (5’-3’) |
| --- | --- |
| qPCR-Hexon F | CCAGAGGCGCAACTTTAT |
| qPCR-Hexon R | ATGTACTCGTAGGTGGTAGG |
| pMD-19T-Hexon F | ATGGCGGCCCTCACGCCCGAC |
| pMD-19T-Hexon R | TTACACGGCGTTGCCTGTGGC |

Table S1 The primers used in the this experiment
